# Local Inequalities in Health Behaviours: Longitudinal Findings from the Stockton-On-Tees Cohort Study

**DOI:** 10.3390/ijerph182111018

**Published:** 2021-10-20

**Authors:** Nasima Akhter, Ross Stewart Fairbairn, Mark Pearce, Jon Warren, Adetayo Kasim, Clare Bambra

**Affiliations:** 1 Department of Anthropology, Durham University, Dawson Building, Stockton Road, Durham DH1 3LE, UK; kasimadetayo@gmail.com; 2 Faculty of Medical Sciences, Population Health Sciences Institute, Newcastle University, Newcastle upon Tyne NE1 7RU, UK; ross.fairbairn@newcastle.ac.uk (R.S.F.); mark.pearce@ncl.ac.uk (M.P.); clare.bambra@newcastle.ac.uk (C.B.); 3 St. Cuthberts Society, Durham University, Durham DH1 3LE, UK; jonathan.warren@durham.ac.uk; 4 Department of Sociology, Durham University, 32 Old Elvet, Durham DH1 3HN, UK; 5 Durham Research Methods Center, Durham University, Durham DH1 3LE, UK

**Keywords:** social determinants, health behaviours, health inequalities, austerity, welfare, social inequality, cohort, survey

## Abstract

This paper provides a longitudinal examination of local inequalities in health behaviours during a period of austerity, exploring the role of ‘place’ in explaining these inequalities. Data from the Stockton-on-Tees prospective cohort study of 836 individuals were analysed and followed over 18 months (37% follow-up). Generalised estimating equation models estimated the deprivation gap in health behaviours (smoking status, alcohol use, fruit and vegetable consumption and physical activity practices) between the 20% most- and least-deprived neighborhoods (LSOAs), explored any temporal changes during austerity, and examined the underpinning role of compositional and contextual determinants. All health behaviours, except for frequent physical activity, varied significantly by deprivation (*p* ≤ 0.001). Smoking was lower in the least-deprived areas (OR 0.21, CI 0.14 to 0.30), while alcohol use (OR 2.75, CI 1.98 to 3.82) and fruit and vegetable consumption (OR 2.55, CI 1.80 to 3.62) were higher in the least-deprived areas. The inequalities were relatively stable throughout the study period. Material factors (such as employment, education and housing tenure) were the most-important and environmental factors the least-important explanatory factors. This study suggests that material factors are the most important ‘place’ determinants of health behaviours. Health promotion activities should better reflect these drivers.

## 1. Introduction

Geographical inequalities in health behaviours (smoking status, alcohol use, fruit and vegetable consumption and physical activity levels) are present at all spatial scales—between neighbourhoods, local authorities, regions and countries [1]. For example, a number of studies have found that there are inequalities in smoking prevalence, with higher rates in places with higher levels of deprivation [2,3,4]. The socio-spatial distribution of alcohol consumption is less clear; whilst some studies have reported that it is associated with area-level deprivation [5], other literature reports an absence of association [6]. A more clear finding is that the most severe levels of alcohol use–and alcohol-related harm—are more prevalent in more-deprived areas. In terms of physical activity, a number of studies have found an inverse association with deprivation [2,5]—people living in more deprived areas have lower levels of physical activity. In terms of diet it is generally recognised that those in more deprived areas consume less fruit and vegetables [6]. 

Geographical research has highlighted the important role that ‘place’ has in shaping these socio-spatial inequalities in health behaviours. In terms of smoking, research has found that areas with a higher density of tobacco retailers have higher smoking rates [7]. Further, the social and spatial stigmatisation of smoking may create areas with higher concentrations of smokers—smoking islands [8]. Associations between higher alcohol consumption and the density of alcohol retail outlets have also been found [9]. Associations with the neighbourhood environment have also been found with regard to physical activity. For example, a study conducted in the USA found that those in less-deprived areas are able to be more physically active as the areas they live in have more facilities available to allow for such activities [10]. Further, residents of areas with a higher density of fast-food outlets have lower rates of fruit and vegetable consumption [11] and food deserts and obesogenic environments increase excessive food intake [12].

These studies draw on the idea that ‘place’ matters for the socio-spatial distribution of health and health behaviour as the characteristics of places can promote salutogenic or pathogenic health behaviors [1]. The definition of place is often contentious [13] but it can be considered as a specific geographical location or area, requiring shared experience and bounded membership [14]. Variations between the characteristics of individuals and their interaction with the social, economic and physical environment thereby combine to shape the nature of a specific place [15]. 

Much of the literature addressing the relationship between health and place conceptualises these mediating factors into two main categories: compositional and contextual. The compositional explanation argues that geographical inequalities in health and health behaviours arise from the individual characteristics of the people that live in the areas, most notably in terms of individual material (e.g., income, housing and employment) and psychosocial (e.g., control and self-worth) circumstances [13,15,16]. On the other hand, the contextual explanation argues that geographical inequalities in health are explained by the characteristics of the local place in which individuals live. This can include factors such as area-level unemployment and access to social and physical resources such as food, health care and green space [2,17,18]. 

A more recent framing of health and place is the political economy one [1,15,19]. It ‘scales-up’ the contextual explanation by highlighting the importance of macro-economics, political choices and public policies—beyond the characteristics of individuals and locales—in shaping both place and health [19,20,21]. A key example of the influences of political economy on health inequalities comes from studies of austerity (a policy response to the 2007/8 financial crisis whereby the UK government—amongst others—reduced public spending and welfare benefits) [22]. The studies found that inequalities in health increased [23,24,25,26]. 

Of course, compositional, contextual and macro political economy factors cannot be viewed independently—they are not mutually exclusive but are relational processes that interact with each other [27]. The characteristics of individuals will often be influenced by the characteristics of their surroundings and societal structures and vice versa. 

This study adds to this important international body of literature by examining the relational nature of contextual, compositional and political economy influences on geographical inequalities in health behaviours through data from the Stockton-on-Tees cohort study. This study examines three main research questions:

1. Are there inequalities in health behaviours between the most- and least-deprived neighbourhoods of Stockton-on-Tees?
2. Which place-based factors are associated with health behaviours?
3. Do any inequalities in health behaviours change temporally during austerity?

## 2. Materials and Methods

**Figure 1:**
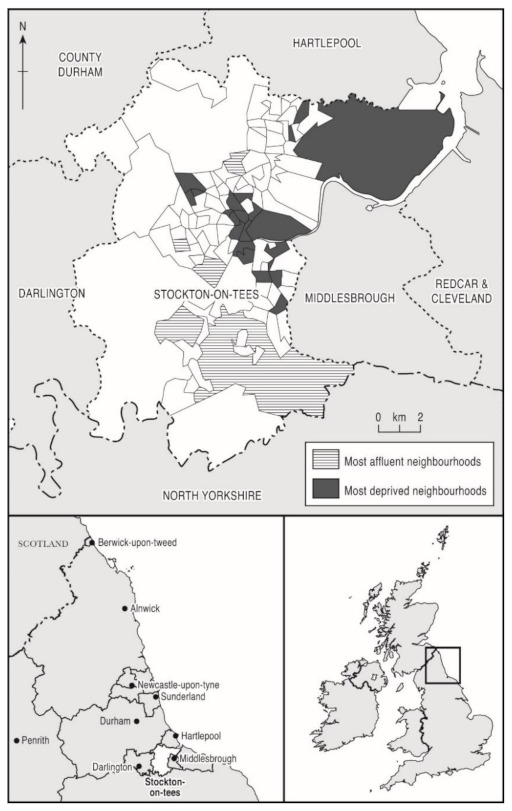
Maps of Stockton-on-Tees showing most- and least-deprived neighbourhoods.

This paper assessed inequalities in health behaviours, factors associated with health behaviours at baseline, and changes in inequality gap over the study period using data from a prospective 18-month household cohort survey of health and the social and behavioural determinants of health. The study was conducted in the most- and least-deprived areas of Stockton-on-Tees, a local authority in the North East of England (Figure 1). Stockton-on-Tees was chosen as the study site because, at baseline, it had the highest neighborhood (lower super output area level (LSOA)) health inequalities in England both for men (a 17-year difference in life expectancy at birth) and for women (11-year gap) [28]. This makes it a particularly important case study for the analysis of health inequalities during austerity. This research is relevant to other local authority areas with similar levels of deprivation and inequality, particularly those in North East England, such as Middlesbrough, Redcar and Cleveland, Gateshead, North Tyneside and Newcastle upon Tyne [29]. Stockton-on-Tees has a population of 191,600 residents [30]. The population is overwhelmingly white (93.4%) [30], and there are high levels of social and economic inequality. 

### 2.1. Sampling and Data Collection

Full details of the sampling technique are contained in Mattheys et al., (2016) [31] and Bhandari et al., (2017) [32]. The sample size was estimated based on a conservative power calculation to detect a 5% difference in health outcomes between the least- and most-deprived areas in Stockton-on-Tees, as measured by validated indicators (EQ5D, SF8 PCS and SF8 MCS) [32]. Allowing for a 20% attrition rate between baseline and first follow-up and an additional 5% attrition at all other follow-ups, an estimated sample size of 800 (400 in each group) was estimated to be required to detect the difference in health between areas. Given the attrition expected, it was assumed that a sample of 800 at baseline would ensure that there would be sufficient participants available in the follow-up period to undertake statistical analysis [31,32]. In summary, the survey used a random baseline sample of adults aged over 18, split between participants from the 20 most- and 20 least-deprived LSOAs of Stockton-on-Tees (derived using 2010 Index of Multiple Deprivation (IMD) scores for England) (Figure 1). Multistage sampling was used whereby the Stockton LSOAs were first grouped into the 20 most- and 20 least-deprived (IMD range 1.54–74.5). Within each group, a random sample of households (addresses) were selected and a single participant per household was determined using a household selection grid to ensure even distribution of age and gender [33]. 

**Figure 2:**
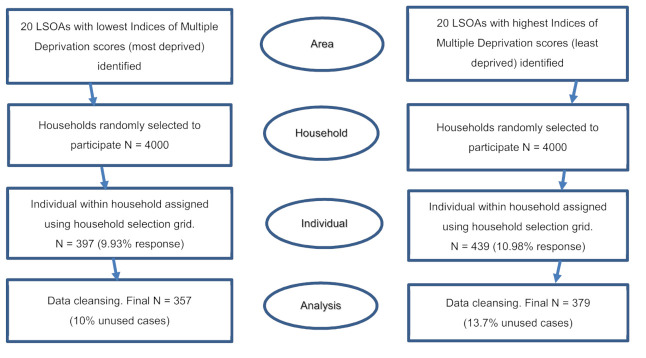
Sampling strategy for the survey [28].

To meet the targeted number of 800 participants, 200 target households were randomly sampled in each of the 40 LSOAs assuming a 10% enrolment rate (because the survey used a postal recruitment approach and so response was expected to be lower than for other recruitment methods) [34,35]. A total of 8000 households (4000 each from the most- and least-deprived LSOAs) were sent study invitation letters to obtain consent to participate in the study based on an opt-in consenting approach. Participants were then surveyed four times over 18 months between April 2014 (baseline, wave 1 and face-to-face) and October 2015 (wave 4 and telephone). Details are presented in Figure 2. This was a baseline response of 10% or 36% of contacted households [31]. Attrition reduced the final wave 4 sample size to 310, a 37% follow-up rate but it fell within our conservative power calculation [31].

### 2.2. Outcome Variables

The questionnaires included questions on mental and physical health, demographics, health behaviours and the social determinants of health. The main outcomes in this analysis are behavioural factors: smoking (yes/no), alcohol consumption (yes/no), fruit and vegetable consumption (five portions per day, yes/no), and physical activity (often, i.e., a couple of times/week, more frequent/not often, or once a week or less). The outcome variables were calculated based on participants’ responses: if they smoke; drink alcohol; how many portions of fruit and vegetables they eat on a usual day; and how often they have physical activity/exercise (everyday, most days, a couple of times a week, once a week, less than once a week, never). Supplementary Table S2 includes the questions asked to collect data on outcome variables.

### 2.3. Explanatory Variables

The primary explanatory variable was area-level deprivation, i.e., whether the participants lived in the ‘least-deprived’ (the 20 LSOAs with lowest IMD scores) or the ‘most-deprived’ (the 20 LSOAs with highest IMD scores) areas within Stockton-on-Tees. Age and gender were used as controlled variables in the models. 

Compositional factors (material) included educational status (highest level of educational qualification achieved), housing tenure (owned outright, rented, mortgaged, rent-free or others), household receipt of benefits (derived from responses on receiving specific listed benefit schemes), receipt of housing benefit (yes, no), employment (currently employed, unemployed), workless household (no adult household members currently in work), and household annual income (recorded via a range of income bands). Psychosocial factors included participants’ perceptions of neighbourhood safety (if they feel safe to walk after dark), lack of companionship (whether lacked companionship: hardly ever, sometimes, often), feeling left out (whether felt left out: hardly ever, sometimes, often), feeling isolated (whether felt isolated: hardly ever, sometimes, often), frequency of social meetings (never, less than once a month, once a month, several times a month, once a week, several times a week, every day), and happiness scale score (range 0–10; 0 = not happy at all, 10 = very happy). 

Contextual factors included whether the respondents reported that the accommodation had problems with damp (e.g., leaking roof, damp wall, rotten wall or floorboard), was dark (at least one room too dark or do not have enough light), was not warm enough (in winter months), or had problems of neighbourhood (within 15-min walk) noise (yes, no), pollution (yes, no) and crime (yes, no). The political economy of place (austerity) was assessed using time. For easier interpretation, categorical variables, such as lack of companionship, feeling left out, or feeling isolated were recoded into binary variables (often = 1, others = 0). Table 1 shows the response categories of the predictor variables.

### 2.4. Statistical Analyses

After data cleaning 736 baseline participants were included within the full analyses: 357 participants from the most-deprived LSOAs and 379 from the least-deprived. At wave 4 follow-up there were 310 participants: 176 from most- and 134 from least-deprived LSOAs. Descriptive analysis of the baseline data was conducted using summary statistics (frequencies, percentages, mean, standard deviation). Generalised estimating equations (GEE) accounting for clustering at LSOA level was applied to the baseline data to quantify the gap in health behaviours and to assess the associations between behaviours and the explanatory factors. To examine inequality in health behaviours, base models were fitted for the behavioural outcomes with only the deprivation indicator as the predictor variable. Thereafter, models including age and sex were fitted to test for associations between behavioural outcomes and deprivation by including explanatory compositional and contextual covariates to obtain a parsimonious model. Since health behaviours tend to vary by age and sex, these variables were included in the models so that the results could be adjusted for presence of these factors. Finally, age- and sex-adjusted models were also used to assess changes in the inequality gap over time (austerity). All statistical analyses were completed on SAS 9.4 version (SAS Institute Inc., Cary, NC, USA).

## 3. Results

### 3.1. Baseline Characteristics

Table 1 provides a descriptive analysis of the baseline sample. It incorporates demographics, behavioural outcomes, and compositional (material and psychosocial) and contextual variables, stratified by deprivation level. For both most-deprived and least-deprived areas, more women than men participated in the study. At baseline, 27.5% of the participants in the most-deprived areas were aged 65 years or over, whilst 32.8% aged 65 years or over were in the least-deprived areas. In addition, a higher percentage of participants living in the most-deprived areas were below 25 years of age (10% vs. 3%). A lower percentage of participants in the most-deprived areas had a degree or higher level of education (5% vs. 27%) than in the least-deprived areas. At least twice as many participants in the most-deprived areas reported feeling isolated, left-out or lacking companionship. However, average happiness score was quite similar in both areas.

In the most-deprived areas, 37.0% of participants smoked, compared to 10.3% in least-deprived areas; about 59.1% of those in the most-deprived areas consumed alcohol, while 78.9% did so in the least-deprived areas. Only 21.6% of participants consumed five or more portions of fruit and vegetables a day in the most-deprived areas, compared to 41.7% in the least-deprived areas; about 67.8% engaged in regular physical exercise in the most-deprived areas, while 60.2% did so in the least-deprived areas. 

### 3.2. Inequalities in Health Behaviours between Areas of Most and Least Deprivation

Most health behaviours varied significantly by deprivation level (Table 2). Smoking behaviour was significantly lower in least-deprived areas (OR 0.21, CI 0.14 to 0.30), whereas, alcohol use (OR 2.75, CI 1.98 to 3.82), and eating five portions of fruit and vegetables a day (OR 2.55, CI 1.80 to 3.62) were significantly higher in the least-deprived areas. However, frequent exercise behavior did not vary significantly by deprivation levels. 

### 3.3. Factors Associated with Health Behaviours

Table 3 contains the final model results about associations of health behaviours with material, psychosocial and environmental factors and Supplementary Table S1 shows the initial bivariate associations tables for the health behaviours. Both tables utilized baseline data only.

The bivariate analysis results found that in terms of material factors, those who were employed, educated and lived in an owned or mortgaged house were significantly less likely to smoke; smoking was significantly higher among those who received housing benefit or any other benefits, or belonged to a workless household. However, alcohol use was significantly more likely among those who were employed, educated (degree or higher) and lived in an owned or mortgaged house. Unlike smoking, alcohol use was significantly lower among those who were in receipt of benefits or belonged to a workless household. Intake of five fruits or vegetables a day and frequent exercise were both significantly higher among those who were educated (degree or higher) or owned their accommodation. However, benefit recipients were significantly less likely to have frequent exercise.

In terms of environmental factors, those who lived in areas with higher levels of noise and crime were significantly more likely to smoke and those living in houses with a dark room were less likely to drink alcohol or consume five portions of fruit and vegetables a day. There were no significant associations between environmental factors and frequent exercise behavior.

In terms of psychosocial factors, those who often lacked companionship, often felt isolated, or were less happy were significantly more likely to smoke. On the contrary, often feeling isolated or left out were negatively associated with alcohol use. Those who socialised more frequently or were happier were also significantly more likely to have five fruit or vegetables a day. Similar associations were seen for frequent exercise.

Overall, more of the material factors than the physical environmental or psychosocial factors were associated with health behaviours.

The final model explaining smoking behavior (Table 3) found that smoking was significantly less in the least-deprived areas, and among those who lived in an owned or mortgaged house compared to those who rented or lived rent-free. However, those who lived in a workless household were significantly more likely to smoke, so were less happy people. On the other hand, the odds of alcohol drinking were significantly higher in the least-deprived areas, among those employed or educated at a degree level (twice as likely for both), but was lower among women. The likelihood of having five portions of fruit and vegetables a day was significantly higher (OR = 2.01; 95% CI = 1.38, 2.95) among those living in the least-deprived areas, having degree level education, or among happier people. 

Women were less likely to exercise frequently (Table 3), and so were those who are employed, received a benefit or belonged to a workless household. The likelihood of doing frequent exercise was significantly higher (OR = 2.65; 95% CI = 1.40, 5.00) among those with a degree level education compared with those having no formal education. Those living in noisy areas had significantly higher likelihood of frequent exercise than those lived in areas that were not noisy. Happiness score was positively associated with frequent exercise.

### 3.4. Inequalities in Health Behaviours over Time

**Figure 3:**
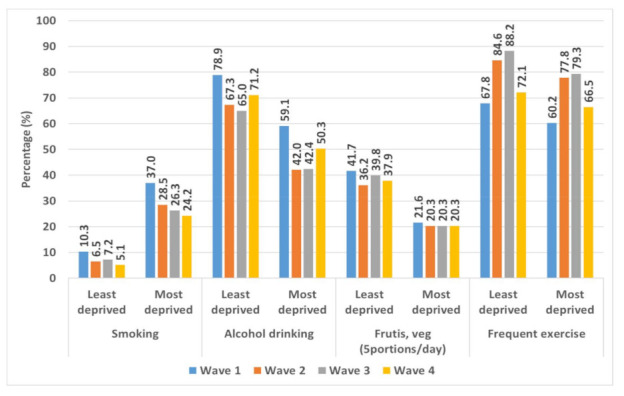
Percentage of respondents smoking, drinking alcohol, eating fruit and vegetables (five portions/day), and exercising frequently in the most- and least-deprived areas of Stockton-on-Tees, over four waves (18 months).

Figure 3 shows the percentage of people practising different health behaviours in the least-deprived and most-deprived areas of Stockton-on-Tees, over the four study waves (18 months). Throughout the period, except for smoking, all health behaviours were more prevalent in the least-deprived areas. Compared to wave 1 (April 2014), the prevalence of smoking was somewhat lower in wave 4 (October 2015) in both types of areas of Stockton on Tees. However, the difference was much smaller (5%) in the least-deprived areas than in the most-deprived areas (13%). The change in prevalence of smoking (wave 4–wave 1) did not vary much between areas (8% and 9% lower prevalence in the least- and most-deprived areas, respectively). Throughout the period, eating five portions of fruit and vegetables a day remained almost two times higher in the least-deprived areas than in the most-deprived areas. The change in prevalence (wave 4–wave 1) was 4% vs. 1% in least-deprived and most-deprived areas, respectively. Contrary to other behaviours, higher percentages of respondents in the least-deprived (4% higher) and the most-deprived (7% higher) areas were having frequent exercise in wave 4.

**Figure 4:**
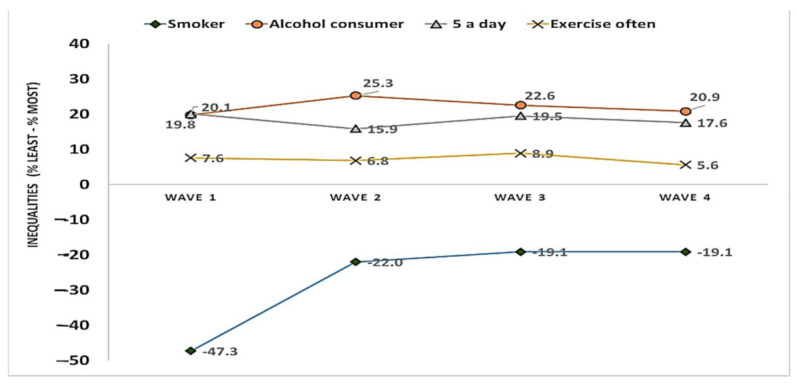
Trends in the inequalities in health behaviours (difference: % least deprived–% most deprived) between most- and least-deprived areas of Stockton-on-Tees, over four waves (18 months).

Figure 4 shows the trends in the inequalities in health behaviours between most- and least-deprived areas of Stockton-on-Tees, over the four study waves (18 months). A considerable inequalities gap (% in least deprived–% in most deprived) persisted across time for each of the health behaviors (Figure 4). Over the study period, the inequality remained much larger for smoking (range: −47% to −19%), alcohol drinking (range: 20–23%), and eating five portions of fruit and vegetables a day (range: 16–20%) than for frequent exercise (6–9%). In terms of variation in inequality between waves, the inequality in smoking became much smaller (−19%) at wave 4 than at wave 1 (−47%). Overall, the variability in inequality between waves was 5%, 4% and 3%, for drinking alcohol, eating five portions of fruit and vegetables a day, and frequent exercise, respectively. It is to be noted that while large inequalities existed in terms of alcohol consumption and fruit and vegetable consumption, between-wave variation for these inequalities was relatively small (Figure 4).

Table 4 shows the results for the GEE model that statistically examined the difference in health behaviours between the least- and the most-deprived areas, and over time (between wave 1 and wave 4). Furthermore, the addition of an interaction term (deprivation status × time) in the model provided the information as to whether the inequality gap had any statistically significant change over the 18 month period. As was observed from the results in Table 2, the model results further confirmed the inequality pattern (less smoking, higher alcohol drinking, and higher consumption of five portions of fruit and vegetables a day in the least-deprived areas than in the most-deprived areas). There was no significant difference in exercise behavior between areas (95% CI: 0.84, 2.87). Although the odds of smoking were significantly lower in wave 4 than wave 1, the odds of smoking remained 80% lower among participants in the least-deprived areas than those from most-deprived areas (OR: 0.20; 95% CI: 0.13, 0.32). However, as the interaction term (deprivation × time) was non-significant, it shows that inequality in smoking behaviour remained stable over the study period. Similarly, the odds of alcohol drinking were nearly three times higher in least-deprived areas (95% CI: 1.84, 3.58), and there was no statistically significant interaction between time and area deprivation. Similar to alcohol drinking, the odds of eating fruit and vegetables (five a day) was about three times higher among those from the least-deprived areas than those in the most-deprived areas. However, there was no statistically significant change in inequality in eating fruits and vegetable over the period (wave 4 95% CI: 0.59, 1.47) than what was observed in wave 1. Unlike other health behaviours, exercise behavior did not vary between the least- and the most-deprived areas and this pattern remained stable over the study period (Table 4).

## 4. Discussion

### 4.1. Main Findings of This Study

This study found that there are inequalities in health behaviours in Stockton-on-Tees. Smoking status, alcohol use and fruit and vegetable consumption all varied significantly by deprivation level. Smoking was more prevalent in the most-deprived areas, while alcohol use, and consuming five portions of fruit and vegetables a day were more prevalent in the least-deprived areas. Our findings about inequality in smoking echo the study by Duncan and colleagues (1999), who used the British Health and Lifestyle survey data to examine inequality in smoking by area and individual factors [36]. They concluded that area-level deprivation has an independent effect on smoking. Similarly, another study from the USA also observed that smoking was highly prevalent among men in more disadvantaged neighbourhoods [4]. A qualitative study to understand area effects on health behaviours in Glasgow, UK also observed associations between smoking and community disadvantage. Living in a disadvantaged area can be more stressful and smoking could be seen as a coping mechanism. Smoking can also represent a cultural norm within low income communities [3]. Unhealthy behaviours tend to cluster in disadvantaged areas—with an increase in area-level disadvantage there is also a tendency for higher rates of multiple unhealthy behaviours [37]. Our findings–that the proportion of participants smoking at wave 4 compared to that of wave 1 dropped largely in the most-deprived areas—could be a result of the disproportionate financial impact of austerity and previous research has found that in times of economic crisis, smoking behavior drops amongst those on low incomes [38,39,40].

In terms of explaining these inequalities, material (compositional) factors were the most- and environmental (contextual) factors the least-important mediators of inequalities in health behaviours, particularly for smoking status and alcohol consumption [3,37,41]. It is also not surprising that large and persistent inequalities existed among our study participants for fruit and vegetable consumption (five portions a day). One study examining pathways of inequality in fruit and vegetable intake in Europe suggested that it can be constrained by financial capability, thus reiterating the association between material factors and health behaviours [42]. Low availability of fruit and vegetables was also found in a USA study after economic change in a neighbourhood following the Great Recession [43]. Social researchers often use the political ecology framework to explain neighbourhood effects on health, which explains how poor political decisions together with ecological factors are associated with persistent structural inequalities and poor health [41]. Material factors were associated with all health behaviours, whereas environmental factors were only associated with frequent exercise. Inequalities in all health behaviours were relatively stable throughout the study period against a backdrop of austerity. Recent research, though, has highlighted that regional inequalities in life expectancy between the North East of England and the South-East, more commonly known as the north–south health divide, is worsening [44]. In this context, our findings of health inequalities in Stockton-on-Tees, an area with long-term exposure to health inequalities calls for priority attention [44,45]. 

### 4.2. What Is Already Known on This Topic

There is extensive international research into the association between deprivation and patterns of health behaviours. Previous research has found that smoking is higher in places with higher levels of deprivation and alcohol consumption can be a means of socialisation and a choice for those able to maintain responsibilities [46] and therefore higher in least-deprived areas, however, severe levels of alcohol use—and alcohol-related harm—is more prevalent in more-deprived areas. There is an inverse association between deprivation and physical activity and diets contain less fruit and vegetables in more deprived areas [2,5,6]. This body of work has also highlighted the important role that ‘place’ has in shaping these socio-spatial inequalities in health behaviours—such as higher access to unhealthy goods and commodities in more deprived communities [7,9,11,12] as well as a lack of health-promoting services and infrastructure such as green spaces for physical activity [10]. Some research has suggested that contemporary austerity has exacerbated health inequalities in England and internationally [23,47], and that people living in the most-deprived areas of England have seen the largest increases in poor mental health [7] and self-harm [8,9,24,25]. 

### 4.3. What This Study Adds

Using a case-study approach, our study has added to this important international body of literature by providing a detailed examination of: (1) geographical inequalities in a range of health behaviours; (2) the relational nature of contextual and compositional factors; and (3) the influence of political economy. It found high inequalities in health behaviours; that material factors were the most- and environmental factors the least-important influences; and that despite austerity, inequalities in all health behaviours were relatively stable throughout the study period. The latter finding is in contrast to previous research into the health impacts of austerity (although in keeping with our own research into physical and mental health) [15,22] and might reflect issues with our sample (older people were largely protected from austerity), the follow-up length (an 18-month follow-up might not have been long enough to detect changes) and the timing of our study (the baseline survey in 2014 was in a period after the economic recession and after some austerity measures had already been implemented) [22]. 

### 4.4. Limitations of This Study

The study is subject to a number of important limitations. The baseline sample size was moderate (although within power calculations) and the response rate was low with only 36% of contacted households (and only 10% of all of our 8000 sampling frame) participating in the survey. The survey also experienced high attrition with only 37% in the final wave [22], some of which could be associated with use of a telephone survey in wave 4. This may undermine the representativeness of the cohort sample and indeed, older people and women were over-represented compared to the general population. Whilst models were adjusted, these factors may still effect the generalisability of the findings. The survey also relied on self-reported health measures, which may have limited precision and reliability. Finally, this study relates only to Stockton-on-Tees. This local authority has the highest gap in life expectancy between people in the most- and least-deprived areas in the whole of England and the results may not be generalisable to other places especially outside of the North East region of England [22].

## 5. Conclusions

This study used a household survey to examine inequalities in health behaviours during a time of austerity. It found clear and stable associations between deprivation and health behaviours. The exploration of risk factors suggests that material compositional factors are the most common determinants of geographical inequalities in health behaviours and that tackling these could be an important approach to health promotion. 

## Figures and Tables

**Table 1:** Baseline descriptive analysis of demographic, material, psychosocial, contextual and behavioural variables in the analysis cohort, stratified by level of deprivation.

Variable		Most Deprived	Least Deprived
Demographics		n (%)	n (%)
Number of participants		357 (48.5%)	379 (51.5%)
Gender			
Male		147 (41.2%)	163 (43.0%)
Female		210 (58.8%)	216 (57.0%)
Age	<25 years of age	37 (10.4%)	15 (3.4%)
	25 to 49 years of age	131 (36.7%)	131 (34.6%)
	50 to 64 years of age	95 (26.6%)	110 (29.0%)
	>65 years of age	94 (26.3%)	123 (32.5%)
Material Socio-Economic			
In paid employment		89 (24.9%)	184 (48.5%)
Educational status	Degree or higher	17 (4.8%)	101 (26.6%)
	Higher diploma/ A level	39 (10.9%)	107 (28.2%)
	GCSE	139 (38.9%)	87 (23.0%)
	Entry level, non-formal	162 (45.4%)	84 (22.2%)
Housing tenure	Own outright	61 (17.1%)	195 (51.5%)
	Mortgage or loan	37 (10.4%)	138 (36.4%)
	Rented	255 (71.4%)	44(11.6%)
	Live rent free	4 (1.1%)	2 (0.5%)
Receiving any benefit		312 (87.4%)	267(70.4%)
Receiving housing benefits		194 (54.3%)	16 (4.2%)
Workless households		238 (66.7%)	143 (37.7%)
Annual income, British pound (mean, SD)		14,244 ± 13,874	35,721 ± 26,227
Material Physical environment			
Damp in home		95 (26.6%)	10 (2.6%)
Home is too dark		63 (17.6%)	31 (8.2%)
Home not warm enough in winter		72 (20.2%)	27 (7.1%)
Neighbourhood noise		86 (24.1%)	42 (11.1%)
Pollution		46 (12.9%)	13 (3.4%)
Crime		105 (29.4%)	24 (6.3%)
Psychosocial			
Neighbourhood safety perception	Very safe	108 (30.3%)	209 (55.1%)
	Safe	132 (37.0%)	141 (37.2%)
	Unsafe	73 (20.4%)	23 (6.1%)
	Very unsafe	44 (12.3%)	6 (1.6%)
Feeling isolated	Hardly ever	256 (71.7%)	312 (82.3%)
	Some of the time	60 (16.8%)	54 (14.3%)
	Often	41 (11.5%)	13 (3.4%)
Feeling left out	Hardly ever	250 (70.0%)	320 (84.4%)
	Some of the time	66 (18.5%)	47 (12.4%)
	Often	41 (11.5%)	12 (3.2%)
Lacking companionship	Hardly ever	241 (67.5%)	288 (76.0%)
	Some of the time	76 (21.3%)	70 (18.5%)
	Often	40 (11.2%)	21 (5.5%)
Happiness scale (mean, SD)		7.41 ± 2.09	7.97 ± 1.61
Frequency of social meetings (mean, SD)		5.43 (1.54)	5.25 (1.35)
Behavioural			
Smoker (yes)		132 (37.0%)	39 (10.3%)
Drink alcohol (yes)		211 (59.1%)	299 (78.9%)
Eat 5 a day fruit and vegetables (yes)		77 (21.6%)	158 (41.7%)
Frequency of physical exercise (often)		215 (60.2%)	257 (67.8%)
Average exercise (min/week, SD)		476.19 ± 545.08	352.49 ± 322.242

**Table 2:** Generalised estimating equation analyses adjusted for age and gender, showing odds ratios, 95% confidence intervals, and *p* values for the association between deprivation and heath behaviours.

Variable	Odds Ratio (OR)	95% CI	*p* Value
Smoking	0.21	(0.14, 0.30)	<0.001
Alcohol use	2.75	(1.98, 3.82)	<0.001
Eating 5 a day	2.55	(1.80, 3.62)	<0.001
Frequent exercise	1.40	(0.83, 2.36)	0.232

**Table 3:** Factors associated with smoking, alcohol use, consumption of five fruits and vegetables/day, frequent exercise, adjusted for clusters, age, gender and deprivation.

Types of Factors	Variables	Category	Smoking	Alcohol Use	Fruits, Veg (5 Portions/Day)	Frequent Exercise
			Odds Ratio (OR), 95% Confidence Interval (CI)			
Socio-demographic	Age		0.96 (0.94, 0.97)	1.00 (0.99, 1.02)	1.02 (1.00, 1.03)	1.00 (0.98, 1.01)
	Sex	Female	1.17 (0.83, 1.65)	0.49 (0.35, 0.69)	1.19 (0.88, 1.66)	0.67 (0.47, 0.93)
		Male	Ref	Ref	Ref	Ref
	Deprivation status	Least	0.63 (0.40, 0.99)	1.82 (1.20, 2.74)	2.01 (1.38, 2.95)	0.96 (0.53, 1.75)
		Most	Ref	Ref	Ref	Ref
Material Socioeconomic (compositional)	House occupancy	Own outright	0.56 (0.35, 0.90)			
		Mortgaged	0.46 (0.25, 0.85)			
		Rent or live rent-free	Ref			
	Educational status	Degree or higher	0.10 (0.04, 0.27)	2.37 (1.40, 4.00)	2.10 (1.19, 3.72)	2.65 (1.40, 5.00)
		A level	0.23 (0.12, 0.47)	1.57 (0.90, 2.72)	1.18 (0.78, 1.77)	1.68 (0.95, 2.95)
		GCSE	0.36 (0.20, 0.65)	1.26 (0.83, 1.90)	1.19 (0.75, 1.89)	1.21 (0.83, 1.76)
		No formal education	Ref	Ref	Ref	Ref
	Employed	Yes		2.24 (1.48, 3.41)		0.41 (0.23, 0.72)
		No		Ref		Ref
	Received benefit	Yes				0.52 (0.33, 0.82)
		No				Ref
	Workless household	Yes	1.69 (1.05, 2.71)			0.50 (0.30, 0.84)
		No	Ref			Ref
Psychosocial (compositional)	Happiness scale		0.87 (0.79, 0.95)		1.10 (1.03, 1.20)	1.17 (1.07, 1.28)
Physical environment (contextual)	Noise in area	Yes				1.69 (1.04, 2.79)
		No				Ref

**Table 4:** Analysis of behaviour outcomes by time and deprivation.

Effects	Categories	Smoking	Drinking Alcohol	Fruits, Veg, 5 Portions/Day	Frequent Exercise
		Odds Ratio (OR), 95% Confidence Interval (CI)			
Age		0.98 (0.97, 0.99)	1.00 (0.99, 1.00)	1.01 (1.00, 1.02)	0.99 (0.98, 1.00)
Gender	Female	1.05 (0.74, 1.49)	0.47 (0.35, 0.52)	1.18 (0.91, 1.53)	0.66 (0.48, 0.91)
	Male	Ref	Ref	Ref	Ref
Deprivation	Least	0.20 (0.13, 0.32)	2.57 (1.84, 3.58)	2.66 (1.85, 3.82)	1.44 (0.84, 2.47)
	Most	Ref	Ref	Ref	Ref
Time	Wave 4	0.62 (0.47, 0.82)	0.48 (0.35, 0.65)	0.85 (0.59, 1.22)	1.27 (0.91, 1.78)
	Wave 3	0.66 (0.47, 0.92)	0.49 (0.40, 0.60)	0.88 (0.64, 1.23)	2.53 (1.46, 4.37)
	Wave 2	0.74 (0.54, 1.01)	0.67 (0.49, 0.92)	0.87 (0.56, 1.36)	2.30 (1.32, 4.01)
	Wave 1	Ref	Ref	Ref	Ref
Time × Deprivation †	Wave 4, Least vs. Most	0.83 (0.47, 1.47)	0.99 (0.63, 1.56)	0.93 (0.59, 1.47)	0.97 (0.61, 1.54)
	Wave 3, Least vs. Most	1.11 (0.67, 1.84)	1.02 (0.78, 1.73)	0.99 (0.63, 1.54)	1.48 (0.74, 2.95)
	Wave 2, Least vs. Most	0.87 (0.50, 1.54)	1.16 (0.78, 1.73)	0.87 (0.51, 1.48)	1.17 (0.57, 2.41)
	Wave 1, Least vs. Most	Ref	Ref	Ref	Ref

^†^ Time and Deprivation interaction term.

## Data Availability

Data available on request.

## Supplementary Materials

The following are available online at https://www.mdpi.com/article/10.3390/ijerph182111018/s1, Table S1: Associations between health behaviours (smoking, alcohol, 5 portions of fruit and vegetables/day, frequent exercise) and material socio-economic, material physical environmental, and psychosocial explanatory variables; Table S2: Description of outcome variables.
