# Supplementary material for: Local Inequalities in Health Behaviours: Longitudinal Findings from the Stockton-On-Tees Cohort Study
Source: Int J Environ Res Public Health. 2021 Oct 20;18(21):11018. doi: 10.3390/ijerph182111018 (PMC8582866; doi:10.3390/ijerph182111018)
Supplement: Supplementary file 1 [file ijerph-18-11018-s001.zip › ijerph-1394057-supplementary.pdf]

Supplementary Materials: The following are available online at www.mdpi.com/article/10.3390/ijerph182111018/s1,

Table S1: Associations between health behaviours (smoking, alcohol, 5 portions of fruits and vegetables/ day, frequent exercise) and material socio-economic, material physical environmental, and psychosocial explanatory variables.

Table S2. Questions for behavioural outcomes

Table S1. Associations between health behaviours (smoking, alcohol, 5 portions of fruits and vegetables/ day, frequent exercise) and material socio-economic, material physical environmental, and psychosocial explanatory variables

Type	Variables	Smoking	Alcohol Use	Fruits, Veg (5 a day)	Frequent exercise
		Odd ratio (OR) and 95% Confidence Intervals CI)			
Material	Employed	0.45 (0.26, 0.76)*	2.34 (1.54, 3.56)*	1.05 (0.76, 1.46)	0.90 (0.59, 1.37)
	Education: Degree or higher	0.09 (0.03, 0.22)*	2.60 (1.55, 4.34)*	2.05 (1.16, 3.62)*	2.67 (1.37, 5.20)*
	Education: Higher diploma/ A level	0.22 (0.11, 0.43)*	1.64 (0.95, 2.83)	1.18 (0.79, 1.78)	1.74 (0.99, 3.06)
	GCSE	0.35 (0.21, 0.60)	1.26 (0.85, 1.87)	1.20 (0.75, 1.90)	1.19 (0.82, 1.74)
	Education: Entry level	Ref	Ref	Ref	Ref
	Tenure : Owned outright	0.37 (0.24, 0.58)*	2.21 (1.46, 3.35)*	1.67 (1.09, 2.56)*	1.67 (1.05, 2.64)*
	Tenure: Mortgaged	0.29 (0.17, 0.49)*	1.73 (1.17, 2.55)*	1.56 (0.92, 2.67)*	1.47 (0.90, 2.39)
	Tenure: Rented, rent free	Ref	Ref	Ref	Ref
	Receiving benefit	1.96 (1.03, 3.74)*	0.52 (0.32, 0.85)*	0.95 (0.64, 1.41)*	0.51 (0.33, 0.78)*
	Income	1.00 (1.00, 1.00)	1.00 (1.00, 1.00)	1.00 (1.00, 1.00)	1.00 (1.00, 1.00)
	Housing benefit	2.14 (1.33, 3.46)*	0.48 (0.35, 0.66)*	0.75 (0.50, 1.13)	0.68 (0.50, 0.93)*
	Workless household	2.26 (1.40, 3.67)*	0.53 (0.37, 0.75)*	1.05(0.76, 1.44)	0.77 (0.54, 1.09)
Physical environment	Too dark room	1.04 (0.67, 1.61)	0.53 (0.34, 0.83)*	0.50 (0.26, 0.96)*	0.81 (0.55, 1.19)
	Damp in house	0.81 (0.53, 1.23)	1.32 (0.83, 2.11)	0.70 (0.43, 1.14)	0.67 (0.41, 1.10)
	Warmth in winter	1.28 (0.81, 2.00)	0.89 (0.58, 1.38)	0.81 (0.46, 1.42)	1.13 (0.78, 1.62)
	Noise in the area	1.54 (1.01, 2.34)*	1.09 (0.70, 1.71)	1.56 (1.06, 2.30)*	1.44 (0.89, 2.31)
	Pollution in the area	1.10 (0.64, 1.89)	0.86 (0.56, 1.30)	1.10 (0.71, 1.69)	1.15 (0.71, 1.88)
	Crime in the area	1.62 (1.10, 2.38)*	0.99 (0.66, 1.47)	0.77 (0.48, 1.24)	1.04 (0.72, 1.51)
Psychosocial	Feel safe	1.31 (0.91, 1.90)	0.71 (0.49, 1.04)	0.73 (0.40, 1.34)	0.65 (0.38, 1.10)
	Often lack companion	2.07 (1.14, 3.74)*	0.83 (0.51, 1.36)	0.85 (0.46, 1.57)	0.69 (0.43, 1.10)
	Often feel left out	1.61 (0.97, 2.68)	0.59 (0.35, 0.99)*	1.04 (0.54, 2.01)	1.04 (0.68, 1.60)
	Often feel Isolated	2.07 (1.20, 3.55)*	0.44 (0.27, 0.79)*	0.80 (0.39, 1.62)	1.15 (0.77, 1.70)
	Social meetings	1.20 (0.97, 1.49)	1.04 (0.84, 1.30)	1.27 (1.03, 1.57)*	1.26 (1.06, 1.50)*
	Happiness scale	0.87 (0.79, 0.95)*	0.96 (0.88, 1.06)	1.09 (1.00, 1.19)*	1.15 (1.05, 1.26)*

*Denotes significant differences. All results are adjusted for age and sex

Table S2. Questions for behavioural outcomes

1. **Smoking**:

Do you smoke?	Yes*	☐
	No	☐

2. **Alcohol drinking:**

Do you drink alcohol?	Yes*	☐
	No	☐

3. **Fruits and vegetables (5 portions/day)*:**

On a usual day, how many portions of fruit and vegetables do you eat? Think of a portion as a large piece of fruit or about a handful of vegetables. (please see SHOWCARD I for example).	number	

4. **Frequent exercise:**

How often do you do exercise/physical activities?	Tick one
Everyday*	☐
Most Days*	☐
A couple of times a week*	☐
Once a week	☐
Less than once a week	☐
Never	☐

*reflects the categories that were considered yes for binary outcomes.
